# Return-to-play criteria based on infrared thermography during anterior cruciate ligament rehabilitation in football players

**DOI:** 10.5114/biolsport.2025.144295

**Published:** 2024-10-23

**Authors:** Victor-Luis Escamilla-Galindo, José Luis Felipe, Antonio Alonso-Callejo, Ralph Van-der-Horst, Ana de la Torre-Combarros, Paolo Minafra, Daniel Fernández-Muñoz, Ismael Fernández-Cuevas

**Affiliations:** 1 Department of research and development, ThermoHuman, Madrid, Spain; 2 INEF, Polytechnic University of Madrid, Madrid, Spain; 3 Performance Analysis Department, UD Las Palmas, Las Palmas de Gran Canaria, Spain; 4 IGOID Research Group, Department of Physical Activity and Sport Sciences, University of Cas-tilla-La Mancha, Toledo, Spain; 5 Physical Therapy and Rehabilitation department of Sports Performance Nederland, Zaanstad, Netherlands; 6 Medical department of Getafe, Madrid, Spain; 7 Medical Department of Modena, Modena, Italy

**Keywords:** Soccer, Rehabilitation, Technology, Injury

## Abstract

Reconstruction of the anterior cruciate ligament (ACL) is associated with degenerative changes during the return-to-play (RTP) process. Infrared thermography, due to its usefulness in monitoring the state of tissues, could help establish physiological criteria for monitoring the knee joint. The aim of the study was to describe normative skin temperature (Tsk) asymmetry of the knee region of interest (knee ROI) from football players during the ACL RTP process. Thirty professional and semi-professional football players, both male and female, from three different European leagues (age: 26.84 ± 3.46 years; height: 1.83 ± 0.22 m; weight: 75.38 ± 5.92 kg) were evaluated using thermography during their RTP process. A linear mixed model was then developed in which Tsk asymmetry was the dependent variable. Sex and Day were included as predictor variables with fixed effects. Athlete was included as a random effect with (Model 1 and Model 2) and without (Model 3) interaction with Day. Model 2 was fitted with a random intercept and random independent slopes, and Model 3 was fitted with a random intercept and random dependent slopes. The results showed the estimates of the model in which both predictor variables were significant. The asymmetry decreased during the days after surgery (-0.01 per day after surgery). The results might help to establish an objective criterion based on infrared thermography for monitoring the rehabilitation stages in the RTP of football players during one year of evolution. The assessment of the thermal asymmetries of the knee ROI during the RTP established a Tsk progression criterion. Medical and technical staff could implement an infrared thermography tool for knee monitoring between RTP stages.

## INTRODUCTION

Professional football teams usually suffer an average of 50 injuries per season [1]. Most football injuries affect the lower limb, with the highest incidence rates (6.8 per 1000 h of exposure) [2]. The knee is the second most injured body region in football, followed by hamstring injury, accounting for 24% of the total injuries among elite football players [3]. The incidence of knee injury in football players is 1.2 injuries/1000 h of exposure [2]. However, in terms of injury burden, which is the product of incidence (the number of times it occurs per 1000 h of exposure) and severity (the days of absence from injury to full return to participation), the knee is the body region with the highest injury burden ratios [4, 5].

Among knee injuries, ACL injury is the most common and severe injury among football players [6]. In addition to that, the economic impact is also significant: previous studies have shown that ACL injury can result in economic losses due to the salary of the players, between €84,000 and €499,000 on average per player from the top 5 European leagues, excluding all other health-related expenses [5].

Thereby, the prognosis of recovering to pre-injury competitive levels indicates that 35%–37% do not reach previous levels [6, 7]. Moreover, the comorbidities associated with ACL injury, such as osteoarthritis of the knee, should also be considered in the process of rehabilitation and in the subsequent sporting life of the football player [8].

In this sense, after ACL reconstruction, a great effort is required to achieve criteria for rehabilitation and return to play (RTP) that will ensure a safe return to sport [9]. Many guidelines have been developed to establish functional criteria for RTP, exercise protocols and strength tests to aid in the rehabilitation process [9–11]. However, none of the previous guidelines have considered the use of infrared thermography as a tool to monitor RTP process. Infrared thermography (IRT) might be considered as an objective alternative to measure internal load. IRT is a non-radiating, contact-free, safe and noninvasive technology that monitors physiological variables through the measurement of skin temperature (Tsk) asymmetry [12]. The use of thermography has been widely studied in construction [13], industry [14], zoology [15], human health [12] and in the world of sport in general [16]. Healthy subjects are supposed to maintain a consistent thermal balance in neutral situations, but the metabolic, biomechanical and physiological demands of the training and competition loads might cause changes in the Tsk from the different body regions (e.g., joints, muscles). Tsk asymmetries in bilateral regions are associated with injury-related factors [17–20]. Measuring Tsk allows for assessing the physiological state of the tissue and establishing criteria for evaluating and monitoring training through to the response of the thermoregulatory system [21]. The measurement of thermal asymmetries has proved to be the most appropriate methodology to monitor the evolution of Tsk in athletes [22, 23].

Therefore, the aim of this study was to describe normative Tsk values from ACL patients that might help to establish a criterion based on infrared thermography for monitoring the rehabilitation stages in the RTP of football players during one year of evolution.

## MATERIALS AND METHODS

## Participants and Inclusion Criteria

A descriptive and longitudinal study was carried out in 30 semiprofessional and professional football players (age: 26.84 ± 3.46 years; height: 1.83 ± 0.22 m; weight: 75.38 ± 5.92 kg; male: 23; female: 7) who were assessed after ACL reconstruction surgery and during at least one year of RTP. Participants were competing in three different professional European leagues (Spain, Italy and Netherlands) before the ACL injury. Data collection was performed at the beginning of the rehabilitation and at the different stages of the RTP, including the average asymmetries of the knee ROI (Table 1). All players participated in a supervised rehabilitation programme performed by medical staff of each team with the aim to return to play. The exclusion criteria were: a) not finishing the rehabilitation process in the place where the evaluation began when being signed by another team; b) not meeting the functional criteria for return to competition in the first year; c) those players for whom data collection or carrying out the exercise programme was not regular; d) players who had at least 8 assessments conducted during the recovery period out of the 9 included in the analysis, meaning they could only skip one assessment in the process. Written informed consent was obtained from both the players beginning the investigation. The current study was approved by the Social Research Ethics Committee of the University of Castilla-La Mancha (Spain) (CAU-732752-Z1C6) and conformed to the recommendations of the Declaration of Helsinki 2013.

**TABLE 1:** Descriptive data of the mean asymmetry of the kneeROI (°C) in ACL-injured football players evaluated with thermography during the return to play (in days post-reconstruction).

Athletes	Gender (Women 0; Men 1)	ACL-reconstruction	Days 2	Days 9	Days 20	Days 35	Days 63	Days 126	Days 187	Days 274	Days 365
User 1	0	10/01/21	2.13	1.88	1.87	0.74	0.96		0.85	0.24	0.38
User 2	1	22/07/20	3.63	2.33	2.11	0.76		0.94	1.53	0.82	0.51
User 3	1	26/12/19		2.53	2.53	0.81	1.21	1.33	1.34	0.76	0.5
User 4	0	16/07/20		2.73	2.4	0.83	2.03	1.56	1.02	0.4	0.43
User 5	1	16/10/20	3.61	2.93	2.38	0.89		1.14	0.7	0.51	0.34
User 6	1	01/12/19	1.64	1.87	1.59	0.93	1.54	0.89	0.65		0.51
User 7	1	29/05/20	2.77	2.11	1.89	0.75	1.99	1.97	0.89	0.8	
User 8	1	01/12/20	1.86	2.1	1.67	1.52	1.09		0.86	0.46	0.45
User 9	1	16/12/20	2.61	3.08	2.39	1.38	2.3	2.01	1.15	0.73	
User 10	1	06/07/20		2.39	3.44	0.74	2.06	1.97	1.2	1.2	0.58
User 11	1	21/05/19	0.93		3.37	0.98	1.24	0.62	0.42	0.58	0.5
User 12	1	25/10/20	3.15	2.89	2.66	1.09	1.85	0.86		0.49	0.51
User 13	1	21/12/20	2.28	1.9		1.57	1.95	1.37	1.05	0.49	0.65
User 14	0	14/01/20	1.28		1.41	2.15	0.97	0.81	0.55	0.54	0.58
User 15	1	27/09/20	2.23	2.89	2.3	1.97	1.64	1.22		0.45	0.45
User 16	1	25/08/20	2.23	2.23		1.05	0.94	0.7	0.65	0.52	0.51
User 17	1	01/08/20	2.29	1.91		0.82	1.3	1.15	0.73	0.62	0.66
User 18	1	15/10/20	3.18	2.76	2.78		1.76	1.18	1.06	1.06	0.56
User 19	0	20/12/20	1.36		3.9	1.34	1.37	1.16	0.87	0.45	0.22
User 20	1	07/12/20	1.06	1.98		0.78	0.62	0.6	0.49	0.6	0.13
User 21	1	02/12/20	2.39	2.21	2.32	1.93	1.46	1.82	0.94		0.54
User 22	1	15/04/19	2.28	2.48	2.75	1.68		1.23	1.3	1.18	0.5
User 23	0	26/01/20	2.72	2.84	2.21	0.95	1.1		0.7	0.41	0.6
User 24	1	27/12/19	2.96	2.97	1.68	0.83	0.99	0.89	0.87	0.56	
User 25	1	26/07/21	3.29		3.23	1.25	1.39	1.33	1.1	1.15	0.6
User 26	1	17/07/20	4.11	2.56	2.12		1.25	1.15	0.86	0.52	0.18
User 27	1	11/12/20	2.8	2.03	1.13	1.74	1.65	1.49	0.79		0.5
User 28	1	03/02/21	2.09	2.13	2.27		1.57	1.29	1.16	0.34	0.45
User 29	0	26/10/21	1.73	2.29		2.13	1.33	0.86	0.96	0.67	0.45
User 30	0	21/03/20	2.13	2.01	2.01	1.87	1.61	0.73	0.67		0.32

## Procedures

### Description of thermography assessment

A thermographic analysis was performed at least 48 h after undergoing reconstruction surgery of the ACL (Day 2). From that moment on, the following thermographic evaluations were carried out up to 9 days later (Day 9), 20 days later (Day 20), 35 days later (Day 35), 63 days later (Day 63), 126 days later (Day 126), 187 days later (Day 187), 274 days later (Day 274), and the last evaluation included up to 365 days after the operation (Day 365). For the thermographic analysis, the same specialist conditioned the room of each club where the thermographic evaluations were recorded, to control the influencing factors under the Thermographic Imaging in Sports and Exercise Medicine (TISEM) consensus [22]. For the evaluation, the thermography specialist used a FLIR T530 thermographic camera (Teledyne FLIR, Oregon, USA) with 320x240 pixels resolution and thermal sensitivity < 0.04°C / < 40 mK. Thermal images from the anterior view of the players’ legs were taken, from which the regions of interest (ROI) were extracted in an automatically and computerized way. The players were instructed to avoid any type of training or treatment before the evaluation.

## Extraction of thermal asymmetries using the software

**FIG. 1:**
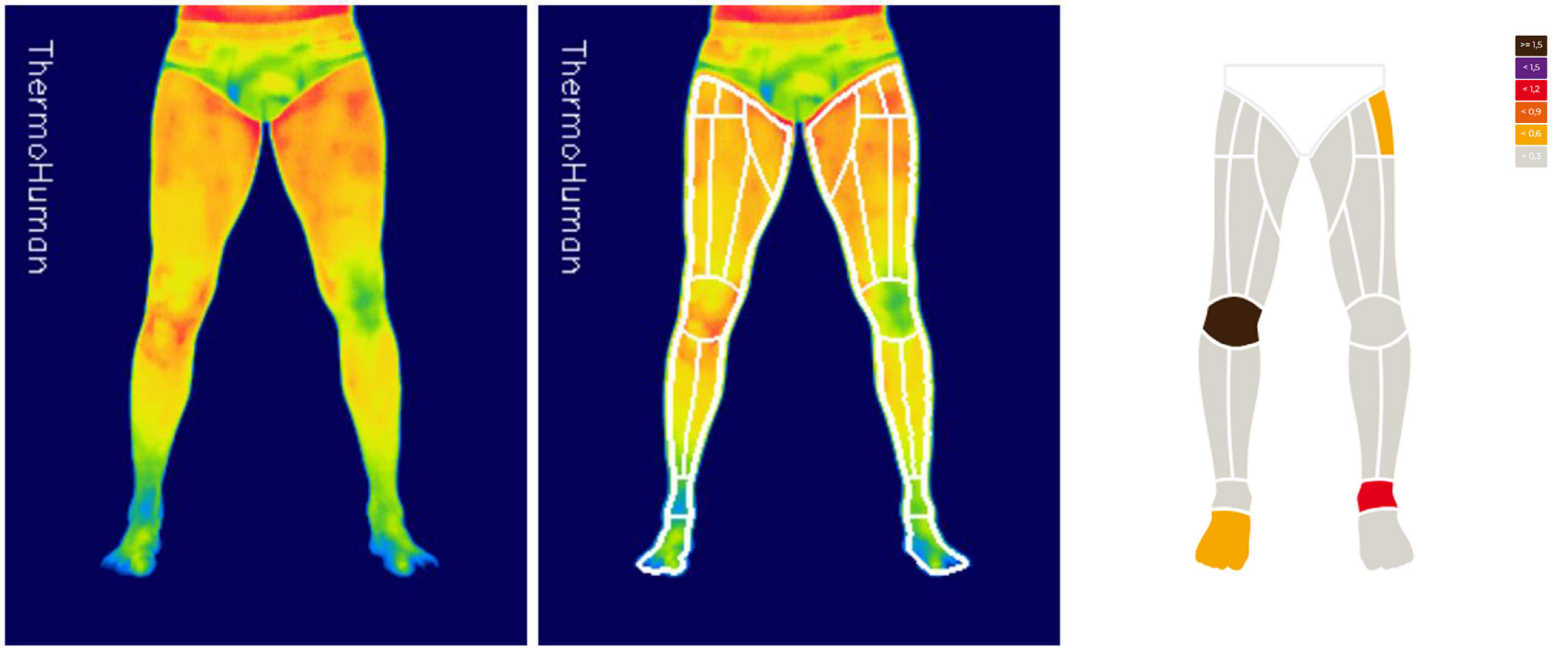
Process of analysis, segmentation, and data extraction from the kneeROI by the software.

For the processing of the thermal images, the ThermoHuman software (PEMA THERMO GROUP S.L., Madrid, Spain) was used. By means of artificial vision algorithms it extracts the segmented regions of interest from the analysed protocols that were previously validated [19], reporting the average, minimum, maximum Tsk and the asymmetry between contralateral regions. For this study, only the knee region (knee ROI) in their anterior view of the legs was analysed (Figure 1).

## Statistical Analysis

Shapiro-Wilk’s and Levene’s tests were used to test the distribution of each group (Day and Sex), confirming normal distribution and homogeneity of variances. A linear mixed model was then developed in which asymmetry was the dependent variable. Gender and Day were included as predictor variables with fixed effects. Athlete was included as a random effect with (Model 1 and Model 2) and without (Model 3) interaction with Day. Model 2 was fitted with a random intercept and random independent slopes and Model 3 was fitted with a random intercept and random dependent slopes.

The final models were displayed as:

Model 1: *y*_ij_ = *β*_0_ + *β*_1_*assesstment*_ij_ + *β*_2_*Gender*_i_ + *ui + v*_i_*assessment*_ij_ + *ϵ*_ij_

Model 2: *y*_ij_ = *β*_0_ + *β*_1_*assessment*_ij_ + *β*_2_*Gender*_i_ + *ui + v*_i_*assessment*_ij_ + *ϵ*_ij_

Model 3: *y*_ij_ = *β*_0_ + *β*_1_*assesstment + β*_2_*Gender + u*_i_ + *ϵ*_ij_

Akaike information criterion was used for selecting the best model. Previously, the presence of the random effect was tested. All statistical analyses were performed in R version 4.2.2 (The R Foundation for Statistical Computing, Vienna, Austria), with RStudio 2022.12.0 using the functions gam() from the package lmer (version 3.1–162) for the model fit.

## RESULTS

**FIG. 2:**
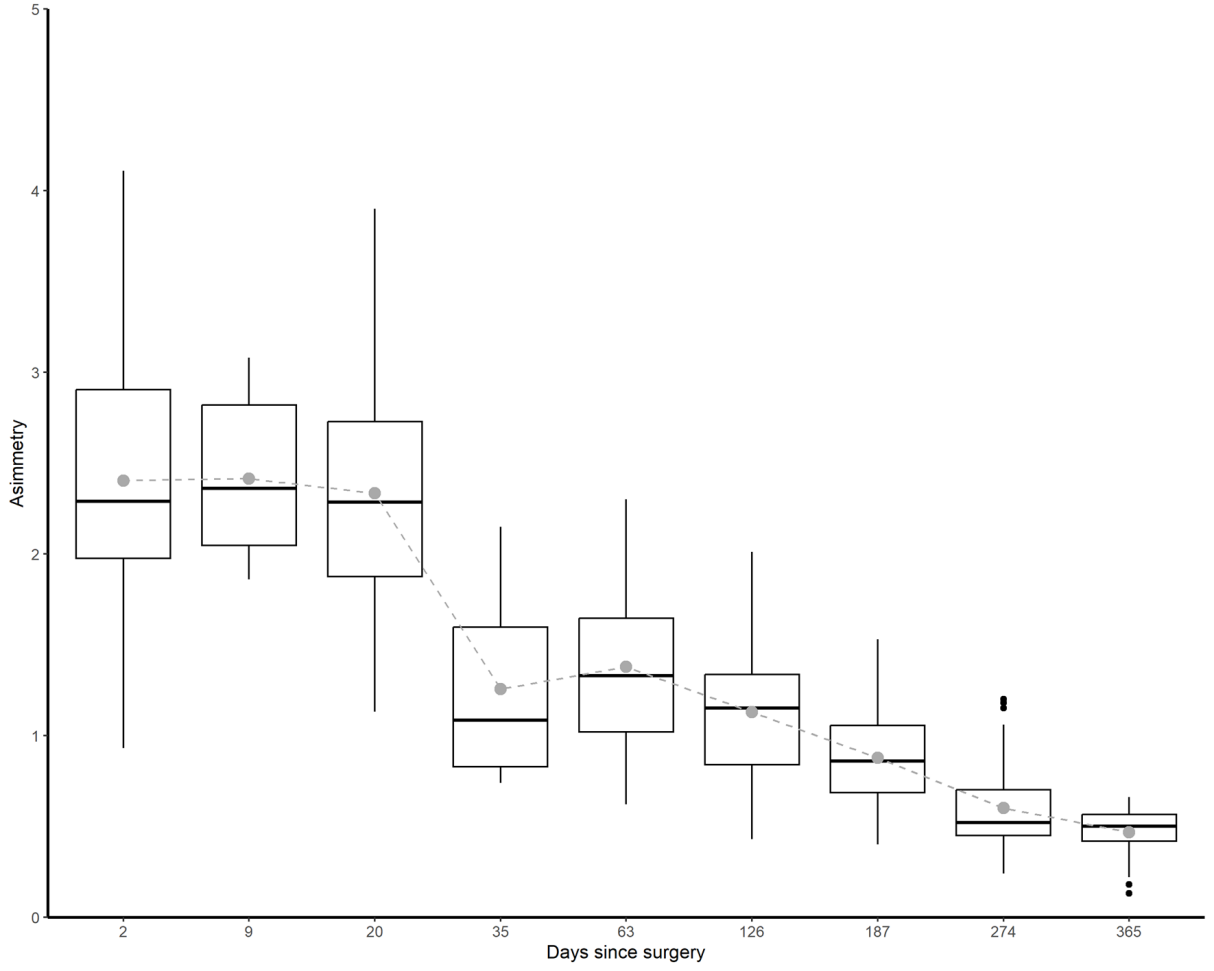
Boxplot by evolution of Tsk asymmetry of injured knee – healthy kneeROI (ºC) during the different assessment stages of RTP (days) process in athletes.

The results revealed a significant reduction in thermal asymmetries from the first stages of rehabilitation to 365 days after the surgery reconstruction (p < 0.01). Mean, median and interquartile range (IQR) of asymmetry values for each Day and its boxplot are shown in Figure 2.

Following the Akaike information criterion (AIC), the selected model was Model 3 (423.57), which showed higher values than the other models (Model 1 = 424.08; Model 2 = 425.57).

Table 2 shows the estimates of the model in which both predictor variables were significant (p < 0.01). The asymmetry decreased during the days after surgery (-0.01 per day after surgery). On the other hand, gender was not a significant variable in the degree of asymmetry generated.

**TABLE 2:** Fixed effects for the linear mixed models

	Estimate	SE	*df*	*t*	*p*-value
(Intercept)	1.92	0.96	47.87	19.98	< 0.01
Day	-0.01	< 0.01	147.40	-17.54	< 0.01
Gender	0.14	0.1	50.05	1.39	0.18

In this model, a football player immediately after surgery might show 2.52°C of asymmetry and 90 days after surgery may show an asymmetry of 1.62°C. However, in the final phases, around the 365^th^ day, there is stabilization of the temperature reduction.

**FIG. 3:**
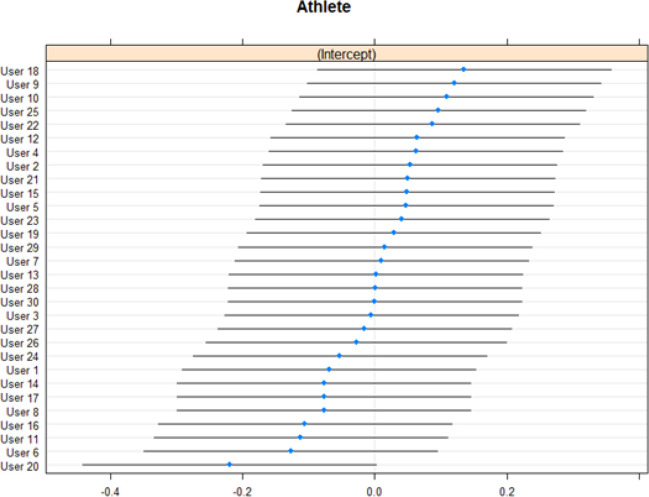
Coefficients for the random effects intercept in the Model 3 for each athlete.

**FIG. 4:**
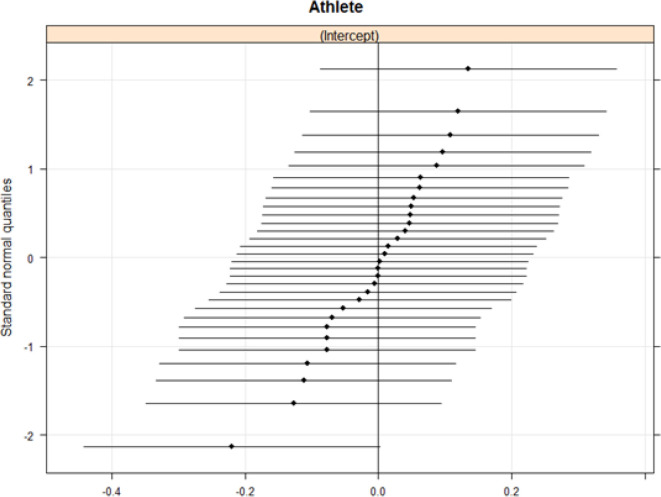
Q-Q plots for the random effects

Figure 3 shows coefficients for the random effects intercept in Model 3 for each athlete and Figure 4 shows Q-Q plots for the random effects.

## DISCUSSION

The aim of this study was to describe changes in the Tsk of the knee ROI throughout the return-to-play process after ACL reconstruction in football players. To the knowledge of these authors, this is the first study to use thermography to describe the evolution of knee temperature according to the chronological stages of the injury in football players. Moreover, it is the first study to establish Tsk throughout their rehabilitation process as one more criterion to achieve a safe RTP.

Managing a return to competition after an injury is one of the issues that most affects professionals involved in sports teams. The RTP after ACL rupture has numerous guides that indicate multilevel criteria by which the football player must evolve for a safe return to sport. These guides range from functional criteria (such as force production or achieving a percentage of less than 10% between the affected leg and the healthy one in horizontal jump metrics) to psychological criteria (the Knee Injury and Osteoarthritis Outcome Score (KOOS)-sport/recreation scale, the International Knee Documentation Committee (IKDC) scale or the Tampa Scale for Kinesiophobia (TSK-11)), where many guidelines are offered to advance from the rehabilitation stage to return to play after ACL reconstruction. The most recent and complete guide, published by the Aspetar group, uses the Grading of Recommendations, Assessment, Development and Evaluation (GRADE) approach to give evidence-based recommendations [9].

Previous studies analysed the rehabilitation process of ACL reconstruction in skiers [24], where differences in asymmetry apparently decreased as the recovery process progressed. Other authors [25] analysed the ACL rehabilitation process in an adult population sample with thermography. These results resemble the outcomes of this investigation. It is important to highlight that in those studies the sample did not consist of professional athletes, who may have greater awareness of problems related to their body and therefore may respond differently [26].

One of the greatest challenges of measuring Tsk is its variability due to technical, environmental and individual factors [12]. In order to minimize the influence of those factors, it is important to rely on standard guidelines [22] and reliable metrics, such as thermal asymmetry [17]. Using absolute Tsk to analyse longitudinal changes over time entails a great exposure to unreliable results due to factors such as the ambient temperature, time of the day or the accuracy of the thermal camera – which normally is ± 2°C [12]. Some research on the use of thermal asymmetries in humans, with very large population cohorts, showed exceptionally low asymmetries when healthy subjects were analysed [27, 28]. However, when thermal asymmetries between two different images taken at different times were analysed, the asymmetry value was higher [28]. Using the methods that have been applied in scientific literature to establish the knee region in thermograms [29, 30], as the software does automatically, and extracting the thermal asymmetry between both sides should become a standard procedure for evaluating the RTP process of the ACL using thermography.

The calculated model predicted a reduction of -0.01°C asymmetry per day after surgery in football players. In relation to this, it is important to bear in mind that during the first week, postoperative inflammation and vascularization of the knee are still very pronounced [31]. Therefore, one of the objectives in these phases is to reduce swelling and inflammation and to be able to recover the gait cycle normally [9]. The aim is to reach day 35 with a reduction in knee inflammation and, consequently, in its temperature.

It may be that between days 35 and 63, there is no significant change in thermal asymmetry between the knees. This could be attributed to the introduction of initial tasks during this period. These tasks involve applying load to the knee and discontinuing the use of orthopaedic aids for walking [9]. This transition may trigger a knee reaction, resulting in the maintenance or slight increase in thermal asymmetry. Furthermore, an adaptation period is required for the knee to accommodate the load during these early rehabilitation phases [9].

There is a progressive trend to reduce knee asymmetry and temperature until the end of the recovery process. Day 187 is one of the dates indicated in the RTP process due to its relationship with graft maturation, functional improvements and assimilation of training loads [9]. In addition, it is one of the milestones in the return-to-play process since many protocols estimate this time as safe for return to competition [9]. Therefore, from this date onwards, thermographic monitoring will be essential for the physiological stress-recovery processes of the ACL-reconstructed knee, with the aim of preventing possible future degeneration and ensuring that the return to play is safe. In this period, there should not be an increase in asymmetry due to inflammatory processes of the knee with ACL reconstruction and the reactions to different stimuli should decrease the asymmetry.

At the end of the process, with an estimated reduction of -0.01 degrees per day, the thermal asymmetry of knee ROI decreased significantly, but it remained elevated above the asymmetry thresholds of healthy knees, with an asymmetry of 0.46 ± 0.13°C. These results are in line with previous research, which described the ‘thermal scar’ as a constant difference on the operated knee [25].

In some research it is pointed out that extending the RTP can favour a safe return after ACL reconstruction [32]. Tsk monitoring of the knee ROI through thermography can help as a criterion for these statements because stabilization of the asymmetry occurs gradually after the surgery. In addition, the value of the asymmetry between the knee ROIs can be a determining criterion to assess joint health in the player’s sports career – especially as the variations in thermal asymmetries between knee ROIs may be related to knee osteoarthritis [33], a common condition in football players with ACL reconstruction [8, 33].

No influence of sex was found, suggesting that these results may be relevant to both male and female football players.

Thermal asymmetry makes it possible to establish objective criteria in the different phases of rehabilitation and return to competition after ACL reconstruction in football players, as well as a series of markers for monitoring Tsk of the knee ROIs in the successive steps of the elite football players’ sports career.

The current study has some limitations. Firstly, no other tests or measurements were collected during the rehabilitation process, and only biological time was considered as a factor of RTP. This is because each club had its own protocol to manage rehabilitation and not all used the same measurements or tools. In addition, the dates of RTP were not collected. Finally, both the level of the leagues and the rehabilitation process were very heterogeneous; each medical service was guided by its protocols and its means of action, which differed between players, and the thermography technician/researcher could only influence the data collection of the knee temperature, being aware of the times since the operation. This is supported by evidence related to the biological maturation time of the plasty, although it was not possible to influence or know the rehabilitation techniques that were used. The performance and physical condition data are the property of the club; the authors only had access and authorization to use the anonymized data of the knee temperature of the athletes.

Despite these limitations, we consider that the study outcomes may be of great utility for medical and technical football staff. They provide an objective criterion through thermal behaviour of the knee to make decisions in order to achieve a safe return to play after an ACL reconstruction.

In future lines of research, the knee ROI Tsk asymmetry values can be combined with those of the front and back thigh Tsk. This study did not include these values, but it has been shown that quadriceps and hamstring strength balance is a key factor to ensure knee stability during the rehabilitation and post-injury period [30]. The combination of knee ROI Tsk asymmetry and quadriceps/hamstring Tsk asymmetry is an interesting research line to explore in the future.

## Practical applications

The assessment of the thermal asymmetries of the knee Tsk during the return-to-play process using infrared thermography is an objective method for evaluation and monitoring between phases. In addition, it allows comparison of the evolution of ACL reconstruction recovery with respect to other professional football players, establishing progression criteria between stages.

The evaluation of the asymmetries described in this study can serve as an objective value in a guide for return to play after ACL reconstruction. Furthermore, as a practical application, medical and technical staff can implement a physiological knee monitoring tool that allows them to assess interventions on the knee throughout the return-to-play process.
